# Observation of Dynamic Cellular Migration of the Medial Edge Epithelium of the Palatal Shelf *in vitro*

**DOI:** 10.3389/fphys.2019.00698

**Published:** 2019-06-06

**Authors:** Gozo Aoyama, Hiroshi Kurosaka, Ayaka Oka, Kohei Nakatsugawa, Sayuri Yamamoto, Safiye Esra Sarper, Yu Usami, Satoru Toyosawa, Toshihiro Inubushi, Yukako Isogai, Takashi Yamashiro

**Affiliations:** ^1^Department of Orthodontics and Dentofacial Orthopedics, Graduate School of Dentistry, Osaka University, Osaka, Japan; ^2^Department of Biological Sciences, Graduate School of Science, Osaka University, Osaka, Japan; ^3^Department of Oral Pathology, Graduate School of Dentistry, Osaka University, Osaka, Japan

**Keywords:** live imaging, cleft palate, craniofacial abnormalities, epithelium, organ culture

## Abstract

Palatal fusion is a critical step during palatogenesis. In this fusing interface, the epithelial sheets need to be removed in order to achieve mesenchymal continuity. Epithelial cellular migration is one of the possible mechanisms, and live imaging of the labeled epithelium could provide direct evidence for it. However, the removal of medial edge epithelium (MEE) between the bilateral processes takes place in the middle of the dorso-ventral axis of the palatal shelf, and thus it is challenging to capture the cellular behavior directly. Here, we evaluate cellular behavior of MEE cells using a live imaging technique with a mouse model which expresses GFP under the promoter of Keratin14 (K14-GFP) and unpaired palatal shelf culture. Using this approach, we successfully obtained live images of epithelial behavior and detected epithelial cell migration on the surface of the secondary palatal shelf without touching of the opposing shelf. Additionally, the pattern of epithelial elimination resulted in oval-shaped exposed mesenchyme, which recapitulated the situation during secondary palate fusion *in vivo*. Detailed image processing revealed that most of the MEE migrated in an outward direction at the boundary regions as the oval shape of the exposed mesenchyme expanded. The migration was preceded by the bulging of MEE, and disappearance of GFP signals was not evident in bulging or migrating MEE at the boundary regions. Furthermore, the MEE migration and the subsequent mesenchymal exposure were disturbed by application of ROCK inhibitor. Together, these findings indicated that epithelial cell migration contributed importantly to the MEE removal and the subsequent exposure of the underlying mesenchyme. Furthermore, they indicated that the migration of epithelial cells was regulated in a time- and space-specific manner, since unpaired palatal shelf culture exhibited these cellular behaviors even in the absence of the opposing shelf. Altogether, present data indicated that this new experimental system combining live imaging with GFP-labeled epithelium mice and unpaired palatal shelf culture enabled direct visualization of cellular migration of MEE *in vitro* and could be a powerful tool to investigate its cellular and molecular mechanisms.

## Introduction

Palatal fusion is a critical step during palatogenesis. The bilateral palatal processes adhere and fuse in the midline to form the secondary palate. In the fusing interface, the medial edge epithelium (MEE) merge to form the epithelial seam, which has to be removed in order to achieve mesenchymal continuity (Ferguson, 1988; Bush and Jiang, 2012). Disruption at any developmental step could result in cleft palate. Many studies have investigated the cellular mechanisms by which MEE disappears from between the two opposed shelves to allow palatal fusion, including mechanisms such as apoptosis, epithelial–mesenchymal transformation, cell migration, and other mechanisms (Hilliard et al., 2005; Dudas et al., 2007). Cell migration was first proposed based on confocal imaging of Dil-labeled cells, which demonstrated that MEE cells migrate orally and nasally to be recruited into the epithelial triangles on both the oral and nasal aspects of the palate (Carette and Ferguson, 1992). *In vitro* chimeric culture using *K14-Cre*; *R26R* mice also revealed that epithelial cell migration is involved in the palatal fusion (Jin and Ding, 2006). However, these issues still remain a matter for debate.

Organ culture of palatal explants serves as a useful tool for studying cellular mechanisms of palatogenesis. Indeed, mouse bilateral palatal shelves successfully fuse even in a cultured condition (Mino et al., 1994). However, the fusing MEE and the epithelial seam intervene between the bilateral processes, and therefore the cellular behavior cannot be directly observed. Interestingly, even if only one side of the shelf is dissected and cultured, MEE cells can be displaced and underlying mesenchymal tissue can be exposed in the absence of contact and adhesion of the opposing MEE (Takigawa and Shiota, 2004; Charoenchaikorn et al., 2009).

In addition, a recent live imaging study using fluorescently labeled transgenic mice provided evidence of dynamic epithelial migration, including an epithelial behavior: “cell protrusion” of the MEE cells, in palatal fusion (Martínez-Alvarez et al., 2000; Kim et al., 2015). This study showed that MEE protrudes to form a cellular bridge for initial contact of the bilateral palatal processes, and then the fused epithelium converges and migrates along the oronasal axis (Kim et al., 2015). However, with progression of the fusion, removal of MEE occurs deep in the intervening region between the bilateral processes and direct observation of the cellular behavior becomes challenging. Recently, confocal microscopy has made it possible to observe the cellular events at various depths in the tissues by using multichannel imaging with Z-stack function. Indeed, cellular tracking using those systems has provided evidence that cellular migration plays critical roles in removal of the MEE cells and palatal fusion. However, the depth of images and data that can be obtained using the confocal microscopy system are limited to approximately 100 um from the superficial layer of the sample (Kim et al., 2017).

Here, we evaluate the cellular behavior of MEE in an unpaired palatal shelf culture using K14-GFP mice (Vaezi et al., 2002). The results demonstrated that the MEE actively migrated during the process of palatal development and resulted in mesenchymal exposure, which expanded with a clear boundary. This method enabled direct observation of MEE cells which were localized in depth by using SEM (Takigawa and Shiota, 2004; Charoenchaikorn et al., 2009; Kim et al., 2015), and time-course observations revealed that occurrence of the MEE removal could be regulated in time- and region-specific manners. Furthermore, ROCK inhibitor disturbed the cellular migration and consequent formation of the oval shape of the mesenchymal exposure. Altogether, our findings show that this experimental model can become a unique tool to enable observation of dynamic epithelial behavior on the medial edge surface of unpaired palatal explants and to explore the possible cellular mechanisms during the displacement of the MEE.

## Results

### Removal of the MEE in Palatal Fusion of K14-GFP Mice

**FIGURE 1:**
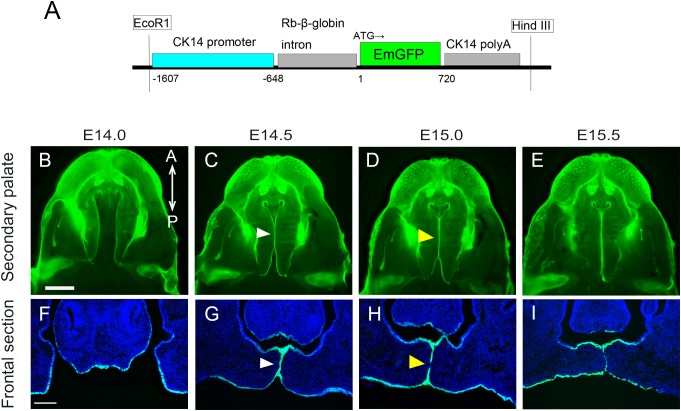
Anatomical and histological analysis of palatal projections during secondary palatal development of K14-GFP mouse. **(A)** The K14-GFP transgene construct. **(B–E)** Fluorescence microscopic image showing oral views of the secondary palate at different developmental stages. **(C–G)** Histological frontal sections from middle regions of the developing palate at each indicated stage. White arrowhead indicates the midline epithelial seam (MES) **(C,G)**. Yellow arrowhead indicates the seam disruption, at which the midline epithelial seam became discontinuous and began to disappear **(D,H)**. A, anterior; P, posterior. Scale bars: **B**, 1000 μm (**B–E** same magnification); **F**, 200 μm (**F–I** same magnification).

In order to confirm the expression of GFP in K14-GFP mice (Figure 1A) during secondary palate development, a series of embryos were analyzed using fluorescent microscopy of both whole-mount and histological sections. Prior to the palatal fusion at E14.0, GFP-positive epithelium covered the entire surface at the medial edge of the palate shelves (Figure 1B,F). At E14.5, the palatal shelves came into contact and formed one GFP-positive epithelial layer (Figure 1C,G, white arrowhead). From E15.0 to E15.5, the region of the contact expanded in both the anterior and posterior directions along the medial edge of the palate, and mesenchymal continuity could be observed (Figure 1D,E,H,I, yellow arrowhead).

The fusion of the bilateral palatal processes into one continuous palate requires integration of MEE overlying the two palatal processes and the subsequent removal of these epithelia to achieve tissue continuity. To evaluate how the fused region expands with time *in vivo*, partially fused bilateral palatal shelves were detached by force and the detached medial edge surface of the mice between E14.0 and E15.5 was directly observed using fluorescent microscopy (Supplementary Figure S1).

Even among mouse embryo littermates at E14.0, there are some variations in growth maturation, and some mice demonstrated only a small region of contact between the bilateral shelves, while some showed more fusion of the palate (data not shown). In these epithelial-GFP labeled mice, GFP-negative cells indicated the exposed mesenchyme, where overlying epithelium had been removed. The initiation of the palatal mesenchymal exposure occurs at the 3rd and 6th rugae regions of the medial edge of the palatal shelf, and both regions expand in both the anterior and posterior directions and also in the oral and nasal directions. At E15.5, mesenchymal continuity was mostly achieved along the entire antero-posterior axis of the secondary palate regions (Supplementary Figure S1). These results clearly show that K14-GFP mice are an appropriate model for tracing the epithelial behavior at the developing secondary palate.

### Ultrastructural Appearance of the Unpaired Palatal Explants

**FIGURE 2:**
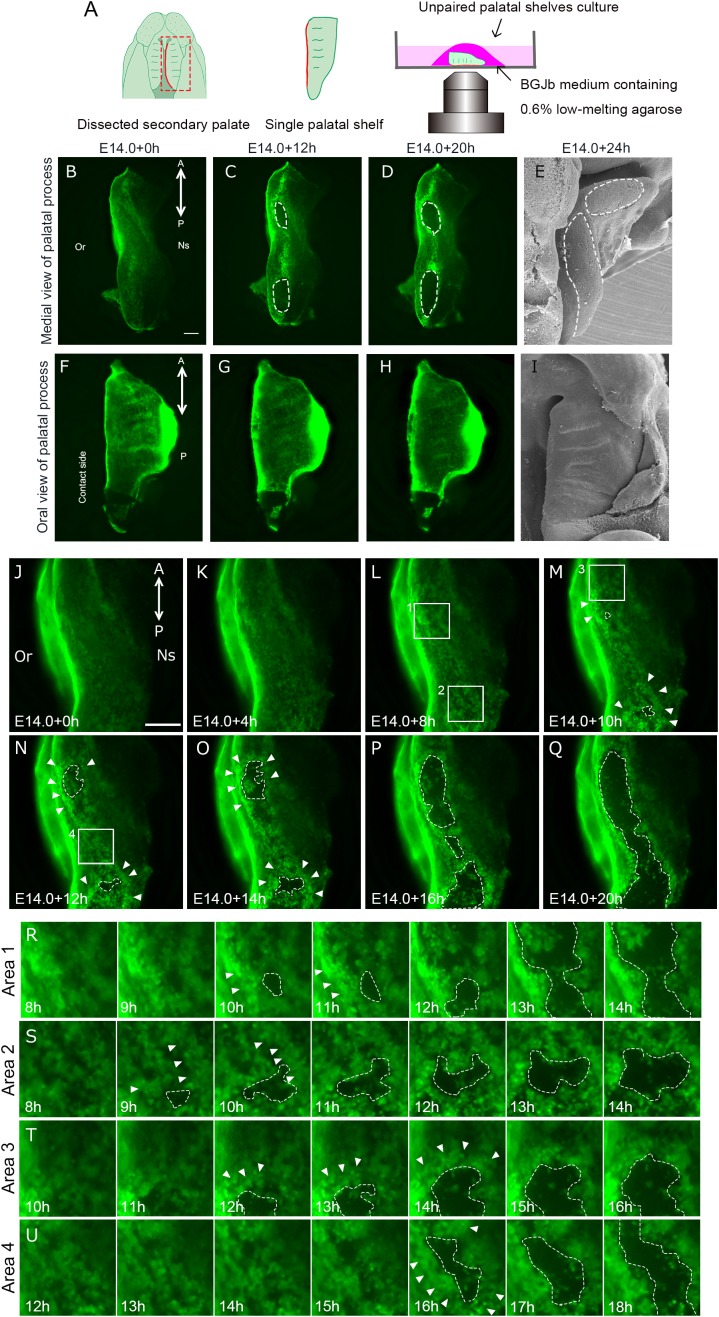
Epithelial behavior in unpaired palatal explant model. And epithelial cell movement captured by live imaging in unpaired palatal explant model. **(A)** Schematic drawing of where the explants located in present experimental model. Fluorescence microscopic images showing the view of medial edges **(B–D)** and the view of the oral side of palatal epithelium **(F–H)** of the unpaired palatal explant model after 0, 12, and 20 h of culture. SEM images showing tissue surface of medial edges **(E)** and oral side **(I)** of unpaired palatal explants after 24 h of culture. White dotted line indicates the area of mesenchymal exposure. **(J–Q)** Time-lapse imaging of K14-GFP mouse unpaired palatal explant cultures reveals the cellular gaps or spaces and bulging in GFP-positive epithelial cells (white arrowheads). White dotted lines indicate the area of mesenchymal exposure. **(R–U)** Higher magnification view of area 1 **(L)**, area 2 **(L)**, area 3 **(M)**, area 4 **(N)**. A, anterior; P, posterior; Or, Oral side; Ns, Nasal side. Scale bars: **B,F**, 200 μm (**B–D** and **F–G**); **J**, 200 μm.

A previous SEM study demonstrated that MEE cells could be displaced when one side of the shelf was removed and unpaired palatal shelf explants were cultured (Takigawa and Shiota, 2004). The palatal shelf was dissected at E14.0 and the explants were cultured either in a humidified cell culture chamber or in an All-in-one fluorescence microscope (Figure 2A). Fluorescent microscopy analysis enabled direct observation of the disappearance of GFP-labeled epithelium and the consequent exposure of the underlying mesenchymal tissue, as also observed in SEM analysis (Figure 2B–D). Such epithelial cell removal was observed at the MEE region but was not evident at any other places such as oral epithelium (Figure 2F–H).

In agreement with a previous SEM study (Charoenchaikorn et al., 2009), our observations of cultured unpaired palatal shelf explants revealed that the region of mesenchymal exposure appeared at two regions around the 3rd and 6th rugae levels, and this exposed region expanded in both the anterior and posterior directions along the medial edge of the palate (Figure 2B–E). Twenty hours after the start of the culture, the oval-shaped mesenchymal-exposure region was further expanded (Figure 2D). In order to further confirm the similarity of this cultured condition and *in vivo* status, partially fused bilateral palatal shelves were detached by force, and the detached medial edge surface of the mice between E14.0 and E15.5, and also the medial edge surface of the detached palate, were directly observed using fluorescence microscopy. As has been described, an oval shaped mesenchymal exposure could be seen in the cultured condition at the position of the 3rd and 6th rugae. This phenomenon could also be seen in the dissected palatal shelf at around E14.25, with scattered GFP expression at a similar position (Supplementary Figure S1, yellow arrowhead), while continuous GFP expression could be seen in the middle of the palatal shelf (Supplementary Figure S1, red arrowhead). Again, these results indicate the similarity of the behavior of palatal epithelium which could be marked by K14-GFP in our experimental conditions. We also observed cultured palatal shelf from the oral side in order to assess whether this mesenchymal exposure is a specific feature which occurs at the site of palatal fusion. The results showed that there was no mesenchymal exposure at the oral side of the palatal shelf even after 20 h of culture (Figure 2F–I). These results indicate that the mesenchymal exposure at the site of fusion in unpaired palatal explants is not an artifact of tissue culture but rather reflects the cellular event of removal of MEE cells during palatal fusion.

### Live Imaging of the Migrating MEE of the Unpaired Palatal Explants

In order to investigate the detailed behavior of MEE cells when the mesenchymal exposure occurs, we performed live imaging using unpaired palatal explants and K14-GFP mice. Until 4 h of culture, the movement of the epithelial cells was subtle and mesenchymal exposure was not visibly evident (Figure 2J,K). After 8 h, bulging epithelium appeared (Figure 2M–O, white arrowheads) and gaps or spaces among the epithelial cells became visible, presumably due to loss of cellular adhesion among the epithelial cells prior to the initiation of MEE removal (Figure 2L,M, white dotted line). From 12 h after starting the culture, the expansion of the area of mesenchymal exposure accelerated together with dynamic epithelial migration (Figure 2N–U, white dotted line, Supplementary Movie S1). Once exposed mesenchyme became evident, live imaging observation clearly demonstrated that most of the epithelium actively migrated outward as the exposed regions expanded, specifically at the boundary between the epithelium and the exposed mesenchyme (Supplementary Movie S2). The GFP-labeled epithelium formed a boundary between the epithelium and mesenchyme, and mass migration rather than individual cell migration appeared to contribute to the expansion of mesenchymal exposure (Figure 2M–U and Supplementary Movie S2). Around the oval regions of the exposed mesenchyme, the MEE cells at the anterior-most region migrated in the anterior direction and those at the posterior region migrated in the posterior direction, with the result that the exposed regions expanded along the anterior-posterior axis. The MEE cells on the oral side of the exposed regions migrated in the oral direction and those on the nasal side migrated in the nasal direction.

Some of the epithelium remained inside the boundary, and such epithelial cells migrated randomly on the exposed mesenchyme (Figure 2R–U). Cell death or sloughing-off behavior could be observed as disappearance of the GFP-signal. In our live imaging, disappearance of GFP-signal was not evident at the boundary between the regressing epithelium and the exposed mesenchyme. Some cells showed disappearance of the GFP label after the migration.

### Cell Proliferation in Mesenchyme in Cultured Palatal Shelves

**FIGURE 3:**
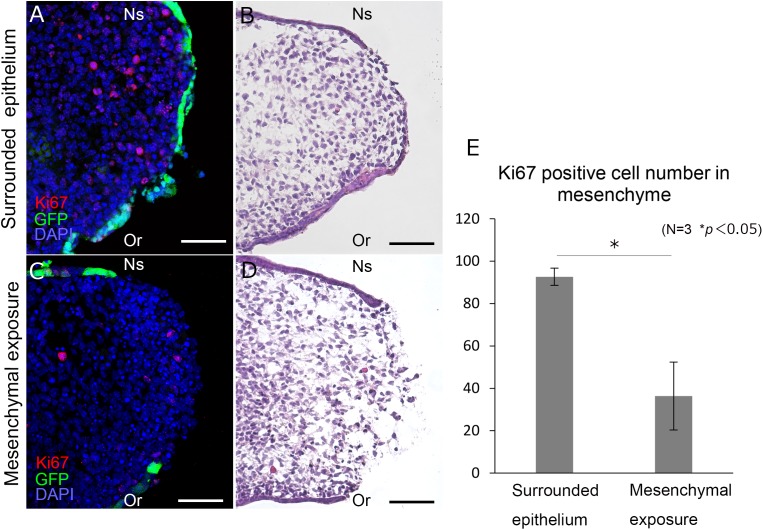
Histological section of the palatal shelf after 12 h unpaired culture. Frontal section of unpaired cultured palatal shelves of K14-GFP mice **(A–D)**. Immunolabeling of Ki67 are shown in different region **(A,C)** with equivalent position of Hematoxylin and Eosin staining **(B,D)**. Statistical analysis of Ki67 positive cells between different regions **(E)**. Ki67 positive cells were counted in a 150 μm × 150 μm area. ^∗^*p* < 0.05, Student’s *t*-test, *n* = 3. Or, Oral side; Ns, Nasal side. Scale bars: **A–D**, 50 μm.

In order to assess the proliferative activity of the mesenchyme in unpaired cultured palatal shelves, histological immunolabeling of Ki67 was performed (Figure 3). Furthermore, the number of Ki67 positive mesenchymal cells were compared between the region with surrounded GFP positive epithelium (Figure 3A,B) and mesenchymal exposure (Figure 3C,D). Interestingly, the number of Ki67 cells were significantly higher in the region with surrounded epithelium (Figure 3E). These results indicate possible differences in mesenchymal proliferative activity in different domain of developing secondary palatal shelves.

### Roles of Rho Signaling in Epithelial Migration of the MEE

**FIGURE 4:**
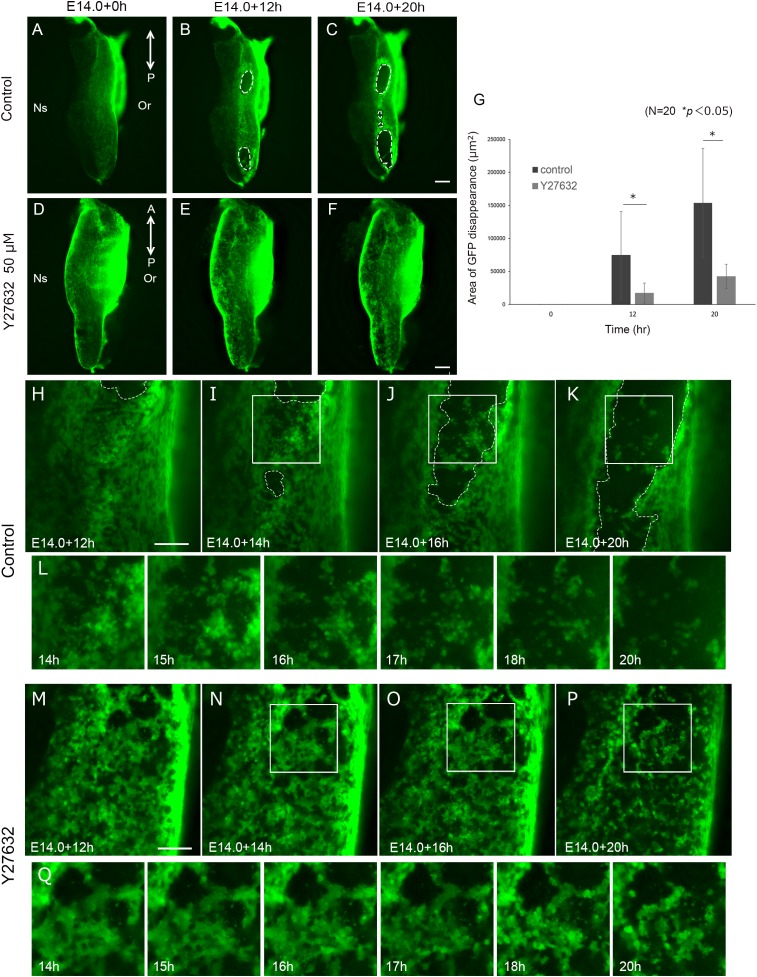
Inhibition of ROCK signaling results in reduction of mesenchymal exposure area. And Inhibition of ROCK signaling inhibited MEE cell migration. Fluorescence microscopic images of K14-GFP mice showing the view of medial edges of the unpaired palatal explant model after 0, 12, and 20 h of culture **(A-C)**. Same stages of palatal shelf which was cultured with ROCK inhibitor Y27632 **(D–F)**. **(G)** The area of mesenchymal exposure was significantly reduced in the group treated with Y27632. ^∗^*p* < 0.05, Student’s *t*-test, *n* = 20. **(H–K)** Time-lapse imaging of K14-GFP mouse using unpaired palatal explant cultures revealed the movement of the epithelial cells and subsequent mesenchymal exposure (white dotted line). **(L)** Higher magnification and detailed time course of live imaging in the region indicated by white boxes in **(I–K)**. **(M–P)** Treatment with ROCK inhibitor Y27632 resulted in less epithelial movement and a smaller area of mesenchymal exposure. **(Q)** Higher magnification and detailed time course of live imaging in the region indicated by white boxes in **(N–P)**. A, anterior; P, posterior; Or, Oral side; Ns, Nasal side. Scale bars: **C,F**, 200 μm (**A–F**, same magnification). **H,M**, 100 μm.

Cellular migration is driven by the reorganization of the actin cytoskeleton (Le Clainche and Carlier, 2008). ROCK is a downstream effector of Rho GTPases that regulate cell migration. In palatal fusion, ROCK inhibitor Y27632 disturbs palatal fusion *in vitro* and live imaging analysis revealed that Y27632 also inhibits MEE migration, indicating that Rho GTPase signaling is essential for the migration of MEE (Kim et al., 2015). These findings also support the idea that migration is important for the removal of the MEE. In order to assess whether the epithelial cellular movement in unpaired palatal culture is controlled by the Rho GTPase signaling pathway, we performed unpaired palatal culture with ROCK inhibitor Y27632. As a result, the oval-shaped mesenchymal exposure which was seen in control palatal shelf cultures was not evident in the cultures with Y27632 even after 20 h (Figure 4A–F). Furthermore, there was a significant reduction of the mesenchymal exposure area in the palatal explants which were cultured with Y27632 (Figure 4G). These results strongly indicate that the epithelial movement which results in the oval shaped mesenchymal exposure is at least partially governed by the Rho-GTPase signaling pathway.

In order to detect the detailed differences of the epithelial cell behavior, we performed live imaging of palatal explants which were cultured with or without Y27632. Interestingly, the movement of the epithelial cells showed a noticeable difference, with low activity in the Y27632-treated explants (Supplementary Movie S3). In addition, the typical oval shape of the mesenchymal exposure (Figure 4H–L) was not observed in the Y27632-treated explants, and some cells remained at the site of palatal fusion even after 20 h of culture (Figure 4M–Q). These results strongly indicated that precise Rho GTPase signaling during palatal fusion is essential for MEE movement which results in mesenchymal exposure.

## Discussion

In palatal fusion, MEE has to be removed for the palate to achieve mesenchymal continuity (Ferguson, 1988; Bush and Jiang, 2012). However, MEE is initially present between the fusing bilateral palatal shelves, and observation of migration of the MEE cells has been challenging. Here, we directly observed the dynamic epithelial cell migration in this region, and the rearrangement of cells on the palatal surface by the combination of live imaging of the GFP-labeled epithelium and the unpaired palatal shelf explant culture system. This unique experimental model provides new tools to observe the behavior of the MEE.

Our live imaging analysis in unpaired palatal shelf culture first detected active migration of the MEE from the medial edge epithelium, which resulted in the formation of the oval-shaped mesenchymal exposure and the further expansion of it. This is in line with the previous finding of *in vivo* palatal fusion and the recent palatal epithelial cell tracking analysis (Kim et al., 2015). However, our direct observation further revealed that epithelial migration occurred in a temporospatially specific manner. In the unpaired palatal culture, the oval shape of exposed mesenchyme expanded with a clear boundary between it and the MEE. In this process, we found dynamic and continuous migration of MEE cells surrounding the exposed mesenchymal surface. Recent cellular tracking analysis provided solid evidence of cellular migration of the MEE in palatal fusion (Kim et al., 2015) and our findings support, at least in part, this finding by direct observation. Our observations showed that the epithelial migration occurred specifically at the region of palatal fusion on the MEE surface, while other regions of epithelium, such as the oral regions remained unchanged. Our live imaging and cellular tracking analysis also demonstrated that most of the MEE cells at the boundary migrated as the region of mesenchymal exposure expanded. Consequently, the exposed mesenchymal surface expanded along the antero-posterior axis and also the oral-nasal axis, while maintaining its oval shape with a clear boundary between the epithelium and mesenchyme. We have also confirmed this mesenchymal exposure by examining histological sections (Figure 3). These findings suggested that the timing and direction of MEE migration was tightly correlated with the expansion of the mesenchymal exposure and regulated in a temporospatially specific manner. The exact mechanism which drives MEE migration is still controversial, and several possibilities have to be discussed. Epithelial cell autonomous migration or passive migration caused by physical pressure from proliferating mesenchyme has been proposed to be part of the mechanism (Takigawa and Shiota, 2004; Chiquet et al., 2016). We also detected mesenchymal cell proliferation in cultured palatal shelves (Figure 3) which implies that part of the driving force of MEE migration in this system is caused by progressive mesenchymal pushes. However, continued research using multiple methods will be required to further reveal the detailed mechanism of MEE migration and disappearance.

Our live imaging also detected some MEE cells at the boundary regions migrated into the exposed mesenchymal region. In contrast to the MEE cells migrating outward, such inwardly directed MEE became dissociated with time and migrated in random directions. It is likely that MEE cells lose cell-cell adhesion and invade adjacent tissues by acquiring cellular motility, as observed in carcinoma cells (Ito et al., 2017). We observed that some MEE cells lost their GFP expression during migration. Since these MEE cells completely disappeared with time as the mesenchymal exposure proceeded in the unpaired palatal culture, it is possible to presume inward-migrating cells have the fate of death during and after migration in our culture system. Indeed, many previous histological studies showed apoptotic activity in the MEE cells in the fusing region of secondary palatal shelves both *in vivo* and *in vitro* (Martínez-Alvarez et al., 2000; Cuervo and Covarrubias, 2004; Takahara et al., 2004). On the other hand, since the stage of mesenchymal exposure in our unpaired culture could correspond to fusing or fused secondary palate, we still do not know if such inward-migrating MEE cells exist in the fusing secondary palate *in vivo*.

The bulging behavior of the palatal epithelium is a specific phenotypic change on the fusing palatal shelves (Fitchett and Hay, 1989; Martínez-Alvarez et al., 2000). Recent live imaging showed that cellular bulges appear in the fusing and migrating MEE cells just before the fusion of the palatal shelves. The present live-imaging demonstrated that the bulging (Martínez-Alvarez et al., 2000) became evident before the MEE started to migrate. It also seemed like once the MEE started to migrate, the bulging occurred as if the region surrounded the exposed mesenchyme. Taken together, our findings are the first to demonstrate that the bulging of the MEE could be associated, at least in part, with subsequent epithelial migration and that this process could be tightly regulated in a temporo-spatially specific manner.

These results show some of the useful features of the present live imaging technique for observing and analyzing epithelial behavior during secondary palate development. It is widely accepted that different live imaging methods each have their advantages and limitations. Since there are still a limited number of live imaging techniques for embryonic secondary palate, continuous investigations aiming to improve existing methods and develop new techniques are important for achieving better observations of cellular behavior during palatogenesis. Since our study also showed that a Rho signaling inhibitor disturbed such epithelial behavior of bulging and migration, the topological cellular arrangement might be important for the phenotypic changes of the fusing epithelium, and the absence of these surface characteristics might impede the fusion of the palatal shelves. For these reasons, our new experimental model which enables direct observation of MEE cells during palatal fusion should be useful for further clarifying the bulging behavior of the MEE.

The unpaired palatal culture system is a unique experimental model (Takigawa and Shiota, 2004; Charoenchaikorn et al., 2009) that enables direct observation of the cellular behavior and phenotypes of the MEE on the tissue surface. Interestingly, in this culture system, MEE cells can disappear from the medial edge of the single palatal shelf independently from palatal shelf contact and midline seam formation. Furthermore, the distribution and timing of this epithelial removal is closely similar to the disappearance of the MEE *in vivo*, as shown in our previous study (Charoenchaikorn et al., 2009). Hence, this experimental model could become a key tool for investigating the cellular behavior in the MEE during palatal fusion. Furthermore, this *in vitro* culture system will enable pharmaceutical and genetic manipulations to study the mechanism of the MEE removal in detail. We are currently crossing K14GFP mice into a mouse model which exhibits cleft palate in order to assess the possible defect in epithelial behavior during cleft palate development. At the same time, we should take into consideration that limitation of this model is that the influence of the opposing epithelium is missing, and therefore it might not reflect the true environment of fusion of the bilateral palatal shelves. It is also true that we never observe mesenchymal exposure at palatal shelves *in vivo* even in cleft palate mouse models. One of the explanations for this discrepancy could be that the BGJB medium that is used in this method does not contain some component(s) necessary for regrowth of the epithelial sheet in the secondary palate after rupture (Takigawa and Shiota, 2007). We should also consider this difference as a limitation of the present unpaired palatal culture system.

## Conclusion

In conclusion, our new experimental system which combines live imaging of the GFP-labeled epithelium and unpaired palatal shelf culture enables direct visualization of cellular behavior of the MEE *in vitro*. Here, we first found that cellular migration contributed importantly to the removal of the MEE and the subsequent mesenchymal exposure in unpaired palatal shelf culture. The bulging of the MEE also preceded the migration, and disappearance of GFP signals was not evident in bulging or migrating MEE at the boundary regions. Furthermore, the MEE migration and the subsequent mesenchymal exposure were disturbed by application of a ROCK inhibitor. Thus, our new experimental system is a powerful tool for exploring the cellular and molecular mechanisms preceding the fusion of the palatal shelves.

## Materials and Methods

### Animals

All of animal experiments were performed in strict accordance with the guidelines of the Animal Care and Use Committee of the Osaka University Graduate School of Dentistry, Osaka, Japan. The protocol was approved by the Committee on the Ethics of Animal Experiments of Osaka University Graduate School of Dentistry (permit number: 26-017-0). We used transgenic mice in which GFP was expressed under the control of the Cytokeratin-14 promoter (K14-GFP) (Vaezi et al., 2002). Mice expressing the transgene were identified by the green fluorescent glow of the skin surface. Mature female mice of C57BL/6J (CLEA, Tokyo, Japan) were mated overnight with a K14-GFP male mouse, and the day on which a vaginal plug was found was designated as day 0 of pregnancy. Time course observation of palatal shelf development was performed by dissecting out K14-GFP mice maxilla from E14.0–E15.5.

### Static Palate Explant Culture

Pregnant mice were euthanized on day 14 of gestation (E14.0) under ketamine (25 mg/kg)/Rompun (8 mg/kg) anesthesia using sterile conditions, and the fetuses were removed from the uterus and placed in BGJb medium (gibco@ Life technologies). Palatal explants were dissected under a dissection microscope as described previously (Charoenchaikorn et al., 2009). Dissected palatal shelves were cultured in a glass bottom dish (Matsunami, Osaka, Japan) with medium containing 0.6% low melting agarose (Wako Osaka, Japan) (Kim et al., 2015). The medium was 500 μl of BGJb supplemented with 100 μg/ml penicillin/streptomycin (Invitrogen). Explants were cultured at 37°C with 5% CO_2_ using a standard CO_2_ incubator for 20 h.

### Histology and Immunohistochemistry

After cultivation, palatal explants were immersion-fixed in 4% paraformaldehyde buffered with 0.1 M sodium phosphate (pH 7.4). These tissues were equilibrated in graded sucrose, and embedded in Tissue-Tek (OCT compound, Sakura). Serial frontal frozen sections (10 μm) from samples were prepared. Cell proliferation was determined using an anti-Ki67 antibody (Abcam), and goat anti-rabbit IgG (Alexa Fluor 488; Invitrogen Life Technologies) was used as the secondary antibody. The number of Ki67 positive cells in the palatal mesenchyme were counted in a 150 μm × 150 μm area.

### Time-Lapse Imaging and Quantitative Analysis

Live images of explant cultures were captured using an all-in-one fluorescence microscope (BZ-X700, Keyence, Osaka, Japan), equipped with filters for GFP (excitation: 475 nm, emission: 525 nm) and DAPI (excitation: 360 nm, emission: 460 nm) channels. The instrument was controlled by the BZ Viewer version 1.0 software of the microscope (Keyence, Osaka, Japan). Time-lapse images were captured with a ×10 0.45 NA objective lens used to collect 57 Z-stacks (8.0 um/step) every 10 min over 8 h. A 0.75 NA objective lens used to collect 38 Z-stacks (6.0 um/step) every 10 min over 8 h, focused on the area where GFP was predicted to disappear. 50 μM Y27632 was added to the medium.

Multiple Z-stack time-lapse images were acquired with BZ-X700 viewer software. The original live imaging (obtained using a ×10 0.45 NA objective) AVI files (960 × 320 pixels, 57 frames) were edited using Photoshop creative cloud 2017 (Adobe, München, Germany). AVI format video files (1920 × 1440 pixels) were converted to mp4 files (1920 × 1440 pixels 48 frames) and analyzed.

Next, in the same way, the original live imaging (obtained using a ×20 0.75 NA objective) AVI files (1920 × 1440 pixels, 48 frames) were edited using Photoshop Creative Cloud 2017. In order to observe cell behavior in detail, the observation range of a 200 μm × 200 μm square was selected as the area where cells migrated.

### Rho Kinase Inhibitor

Rho kinase inhibitor Y27632 (SIGMA-ALDRICH^®^) was added to the culture medium at 50 uM to block the ROCK signaling pathway. In order to examine the cell migration inhibitory effect, the area of the region of the palatal shelf where GFP disappeared during culturing was measured using Photoshop Creative Cloud 2017.

### Statistical Analysis

The statistical analyses were performed as indicated in the text. The differences among means were evaluated by Student’s *t*-test. *P*-values < 0.05 were considered to indicate statistical significance.

## Author Contributions

GA performed the experiments and wrote the manuscript. HK funded the study, performed the experiments, and wrote the manuscript. AO, KN, SY, SS, YU, TI, and YI performed experiments. ST provided technical support and conceptual advice. TY supervised the experimental analysis, edited the manuscript, and supplied funds. All authors discussed the results and implications and commented on the manuscript at all stages.

## Conflict of Interest Statement

The authors declare that the research was conducted in the absence of any commercial or financial relationships that could be construed as a potential conflict of interest.

## Supplementary Material

FIGURE S1 GFP expression in developing palatal shelf in K14-GFP embryos. **(A–E)** Oral views of the secondary palate at different developmental stages, **A**, **C–E** in the figure is copied from Figure 1A–E. Fluorescence microscopic images showing medial views of dissected palatal process at progressive developmental stages **(F–J)**. Red arrowhead indicates the position of the first contact, and yellow arrowhead indicates the scattered GFP expression area. White dotted line indicates the area of mesenchymal exposure. A, anterior; P, posterior; Or, Oral side; Ns, Nasal side. Scale bar: **A**, 1000 μm; **F**, 200 μm (**F–J** same magnification).

MOVIE S1 Medial edge epithelium removal by dynamic epithelial migration. Live imaging of medial edges of unpaired palatal explant model shows a series of processes in which cellular gaps or spaces appear and the area of mesenchymal exposure expands. Scale bar: 100 μm. Images were captured every 10 min for 20 h.

MOVIE S2 Expansion of the area of mesenchymal exposure by dynamic epithelial migration. Live imaging of medial edges of unpaired palatal explant model shows that most of the epithelium actively migrates in both the oral side and the nasal side around the exposed mesenchyme. Scale bar: 50 μm. Images were captured every 10 min for 8 h.

MOVIE S3 Inhibition of ROCK signaling inhibited epithelial cell migration. Live imaging of medial edges of unpaired palatal explant model treated with 50 μM Y27632 showed retarded MEE cell movement with inhibition of oval mesenchymal exposure. Scale bar: 50 μm. Images were captured every 10 min for 8 h.
