# Supplementary figures and images for: Observation of Dynamic Cellular Migration of the Medial Edge Epithelium of the Palatal Shelf in vitro
Source: Front Physiol. 2019 Jun 6;10:698. doi: 10.3389/fphys.2019.00698 (PMC6562562; doi:10.3389/fphys.2019.00698)

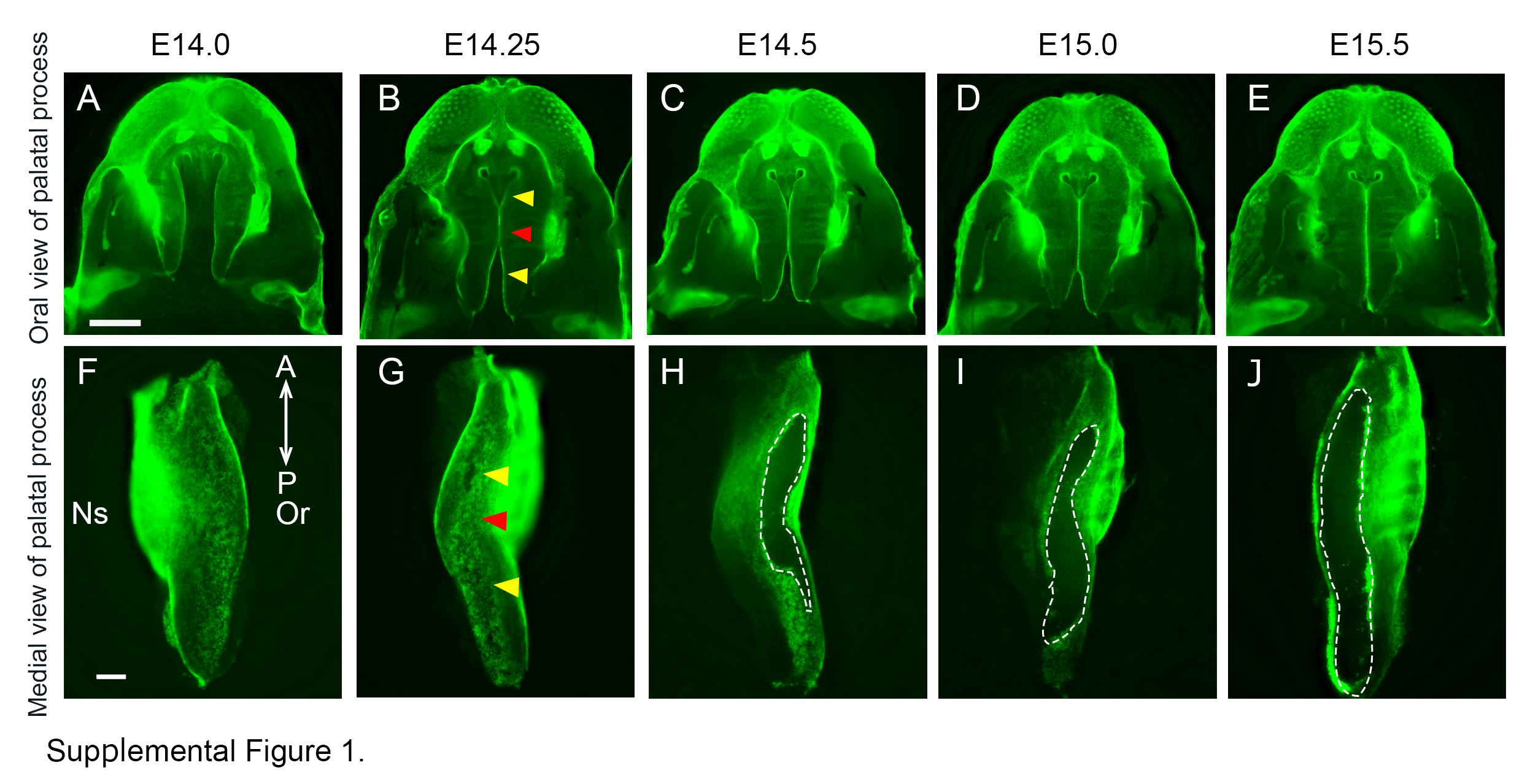

Supplement: FIGURE S1 — GFP expression in developing palatal shelf in K14-GFP embryos. (A–E) Oral views of the secondary palate at different developmental stages, A, C–E in the figure is copied from Figure 1A–E. Fluorescence microscopic images showing medial views of dissected palatal process at progressive developmental stages (F–J). Red arrowhead indicates the position of the first contact, and yellow arrowhead indicates the scattered GFP expression area. White dotted line indicates the area of mesenchymal exposure. A, anterior; P, posterior; Or, Oral side; Ns, Nasal side. Scale bar: A, 1000 μm; F, 200 μm (F–J same magnification). [file Image_1.TIF]
